# Ultrasound-Guided Axillary Management in Early-Stage Breast Cancer: From Diagnosis to De-Escalation

**DOI:** 10.3390/cancers18152405

**Published:** 2026-07-26

**Authors:** Xiangmin Shen, Zi Yi, Kui Tang

**Affiliations:** 1 Department of Neurology, The Second Xiangya Hospital, Central South University, Changsha 410011, China; sxm010001@126.com; 2 Department of Cardiovascular Surgery Intensive Care Unit, The Second Xiangya Hospital of Central South University, Changsha 410011, China; yizi1688@163.com; 3 Department of Ultrasound Diagnosis, The Second Xiangya Hospital of Central South University, Changsha 410011, China; 4 Research Center of Ultrasonography, The Second Xiangya Hospital of Central South University, Changsha 410011, China

**Keywords:** breast cancer, axillary lymph node, ultrasound, sentinel lymph node biopsy, de-escalation therapy, neoadjuvant chemotherapy, targeted axillary dissection

## Abstract

Axillary lymph node management in early-stage breast cancer has undergone a fundamental transformation over the past three decades, shifting from routine radical clearance to precision-guided de-escalation. Ultrasound (US) has emerged as the central imaging modality in this paradigm shift, serving as the first-line tool for preoperative nodal assessment, guidance for biopsy and clip placement, monitoring of response to neoadjuvant chemotherapy (NAC), and localization of marked nodes for targeted axillary dissection (TAD). This review systematically examines the evolution of axillary management in early-stage breast cancer through a US-centric lens. We summarize the historical transition from Halstedian axillary lymph node dissection (ALND) to sentinel lymph node biopsy (SLNB), critically appraise the landmark trials—Z0011, AMAROS, OTOASOR, and SOUND—that have progressively de-escalated axillary surgery, and detail the role of US in preoperative staging (including conventional B-mode, superb microvascular imaging, contrast-enhanced US, elastography, and AI-assisted diagnostics), US-guided biopsy and node marking, and post-NAC TAD. We further explore the integration of US with liquid biopsy, multigene profiling, and molecular subtype-guided systemic therapy to enable individualized decision-making. Finally, we propose a stepwise US-centric clinical algorithm and discuss future directions, including AI-powered real-time interpretation, imaging-omics, and US-guided targeted therapy. This review provides a comprehensive framework for incorporating ultrasound as a key component of multidisciplinary decision-making—alongside pathological, surgical, radiotherapeutic, and molecular inputs—in contemporary axillary management.

## Simple Summary

Breast cancer is the most common cancer in women worldwide. An important part of treatment is assessing whether cancer has spread to the lymph nodes under the arm. In the past, doctors routinely removed all these lymph nodes, which often caused arm swelling and other side effects. Today, ultrasound imaging helps doctors see which lymph nodes are suspicious, guide biopsies, and plan less invasive surgeries. This review explains how ultrasound has become a key tool in modern breast cancer care, allowing many patients to avoid extensive surgery while still achieving excellent cancer control. We summarize the major clinical trials that have made this possible, discuss new ultrasound techniques and artificial intelligence, and propose a practical step-by-step guide for doctors to use ultrasound in everyday practice. This review is intended for clinicians, researchers, and anyone interested in understanding how breast cancer surgery has evolved to become more precise and less harmful.

## 1. Introduction

Breast cancer is the most common malignancy among women worldwide, with over 2.3 million new cases diagnosed annually according to the 2022 GLOBOCAN estimates. Axillary lymph node status has long been recognized as one of the most important prognostic factors in breast cancer, directly informing staging, adjuvant treatment decisions, and long-term survival outcomes [1]. Since Halsted introduced radical mastectomy with en bloc axillary lymph node dissection (ALND) in the late 19th century, axillary surgery has remained a central pillar of breast cancer management [2]. For nearly a century, ALND was regarded as the gold standard for achieving regional control through complete removal of axillary lymphatic tissue.

However, the epidemiological landscape of axillary lymph node metastasis has shifted substantially in recent decades. The widespread adoption of early screening programs, improvements in systemic adjuvant therapy, and—crucially—the evolution of high-resolution ultrasound for preoperative nodal evaluation have collectively reshaped clinical practice. An increasing proportion of patients now present with clinically node-negative (cN0) disease. The introduction of sentinel lymph node biopsy (SLNB) in the 1990s marked the dawn of a minimally invasive era in axillary surgery [3]. Subsequent landmark clinical trials—including Z0011, AMAROS, OTOASOR, and SOUND—gradually challenged the necessity of conventional ALND in selected patient populations, driving a paradigm shift from maximal resection toward minimally necessary intervention [4,5].

**Figure 1:**
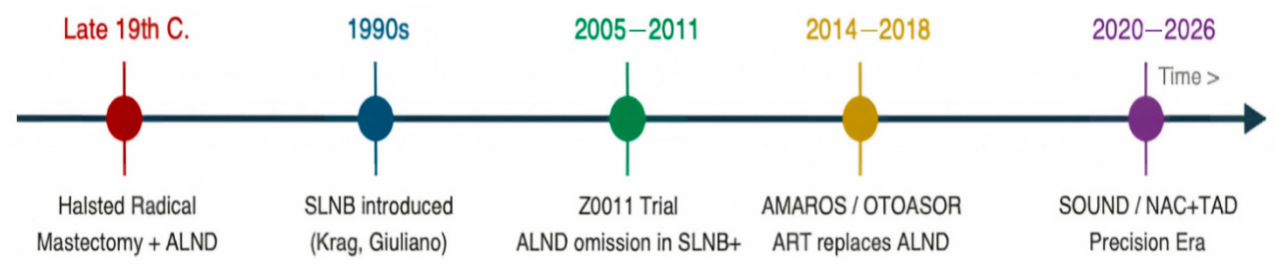
Evolution of axillary lymph node management in breast cancer–a timeline of landmark trials. This chronological timeline illustrates the progressive paradigm shift from routine radical resection (Halstedian ALND) to precision-guided, ultrasound-enabled de-escalation. Key milestones are mapped from the introduction of SLNB in the 1990s, through the landmark de-escalation trials (Z0011, 2005–2011; AMAROS, 2014–2018; SOUND, 2020–2026), towards future precision-guided approaches incorporating AI, ctDNA, and molecular subtyping. ALND = Axillary Lymph Node Dissection; SLNB = Sentinel Lymph Node Biopsy; ART = Axillary Radiotherapy; NAC = Neoadjuvant Chemotherapy; TAD = Targeted Axillary Dissection.

Ultrasound has emerged as the indispensable anchor modality in this transformation. It is now the first-line imaging tool for axillary assessment, guiding every step of the clinical pathway: from initial triage and biopsy guidance, through clip placement before neoadjuvant chemotherapy, to post-treatment response evaluation and targeted axillary dissection. Sonographic assessment detects morphological abnormalities suggestive of nodal metastasis, including cortical thickening, loss of the fatty hilum, rounded shape, and irregular margins [6]. Advanced ultrasound techniques—including superb microvascular imaging (SMI), contrast-enhanced ultrasound (CEUS), shear-wave elastography (SWE), and artificial intelligence (AI)-assisted diagnostics—are further refining preoperative risk stratification [7]. Concurrently, the widespread use of neoadjuvant chemotherapy (NAC) in node-positive patients has enabled targeted axillary dissection (TAD) and clipped-node techniques, advancing the frontier of individualized axillary management [8]. The evolutionary trajectory of axillary lymph node management, from Halstedian ALND to future precision-guided de-escalation, is illustrated in Figure 1.

## 2. Literature Search Strategy

To ensure transparency and reproducibility, we conducted a systematic literature search following the Preferred Reporting Items for Systematic Reviews and Meta-Analyses (PRISMA) guidelines. We searched PubMed, Web of Science, and Scopus databases for articles published between January 2011 and June 2026, using the following search terms: (“breast cancer” OR “breast carcinoma”) AND (“axillary lymph node” OR “axillary staging” OR “sentinel lymph node biopsy” OR “axillary lymph node dissection”) AND (“ultrasound” OR “sonography” OR “contrast-enhanced ultrasound” OR “elastography” OR “artificial intelligence”) AND (“de-escalation” OR “neoadjuvant chemotherapy” OR “targeted axillary dissection”). We included original research articles, systematic reviews, meta-analyses, and major clinical trials (Z0011, AMAROS, OTOASOR, SOUND, INSEMA, Z1071) published in English. Case reports, conference abstracts, and non-peer-reviewed preprints were excluded. Reference lists of retrieved articles were manually screened for additional relevant studies. A PRISMA flow diagram is provided as Supplementary Figure S1.

## 3. The Pivotal Role of Ultrasound in Multidisciplinary Axillary Assessment

### 3.1. Ultrasound as the First-Line Imaging Modality for Axillary Staging

Ultrasound is the primary imaging tool for preoperative axillary evaluation due to its wide availability, low cost, real-time capability, and absence of ionizing radiation. The sensitivity of axillary ultrasound for detecting metastasis ranges from 50% to 85% across studies, depending on disease prevalence, operator experience, and diagnostic criteria. A meta-analysis of 38 studies reported a pooled sensitivity of 79% and specificity of 78%, with a negative predictive value (NPV) of 82% [6]. With the introduction of SMI, CEUS, and other advanced techniques, specificity in contemporary practice has improved further [7].

The most widely accepted sonographic criterion for suspicious axillary nodes is cortical thickness exceeding 3 mm, with loss of the fatty hilum also strongly associated with metastasis [6,7]. Importantly, the negative predictive value of axillary ultrasound is sufficiently high to triage patients effectively. Those with a normal ultrasound can safely proceed to SLNB, whereas those with suspicious nodes undergo ultrasound-guided fine-needle aspiration (FNA) or core biopsy. This approach reduces unnecessary SLNB procedures in patients who are already node-positive, thereby improving preoperative staging accuracy and facilitating appropriate neoadjuvant or upfront surgical planning [6]. Studies have shown that routine axillary ultrasound followed by selective biopsy can identify approximately 40–60% of node-positive patients before surgery, sparing them a separate SLNB procedure and enabling upfront multidisciplinary treatment planning [7].

### 3.2. Ultrasound-Guided Biopsy and Node Marking

For patients with suspicious axillary nodes on ultrasound, ultrasound-guided FNA or core needle biopsy constitutes the standard of care. The biopsied node is routinely marked with a clip or carbon tattoo at the time of biopsy. This “mark then treat” strategy—enabled entirely by ultrasound—is the cornerstone of modern axillary management in the neoadjuvant setting [8,9]. The marked node serves as the target for subsequent targeted axillary dissection (TAD) after NAC, allowing surgeons to accurately excise the previously biopsied node even if it has become sonographically inconspicuous following treatment. This approach is now recommended by international consensus guidelines [8].

A meta-analysis of 26 studies evaluating image-guided localization of clipped nodes after NAC reported a pooled detection rate of 95% and a false-negative rate of 6.2% for TAD [10]. When TAD was combined with SLNB (dual localization), the false-negative rate decreased to 4.2%, meeting the prespecified acceptable threshold of <5% [8]. These results underscore the critical importance of ultrasound not only for initial diagnosis but also for the entire sequence of nodal management in the neoadjuvant setting.

### 3.3. Advanced Ultrasound Techniques: Beyond B-Mode

The following advanced ultrasound techniques are presented in descending order of current clinical readiness: SMI and CEUS are classified as emerging (promising but requiring further standardization); SWE is classified as exploratory (limited multicenter validation); AI-assisted diagnostics are considered investigational. A comparison of these techniques is provided in Table 1.

#### 3.3.1. Superb Microvascular Imaging (SMI)

Superb Microvascular Imaging (SMI) is an advanced Doppler technique that suppresses tissue motion artifacts while preserving low-velocity flow signals, enabling visualization of microvessels. In axillary lymph node assessment, SMI has demonstrated promise in distinguishing metastatic from benign nodes by evaluating intranodal microvascular architecture. Metastatic nodes frequently exhibit peripheral or mixed vascularity with chaotic branching patterns, whereas benign nodes tend to show hilar or central vascularity with orderly branching. A prospective study found that SMI increased the specificity of axillary ultrasound from 72% to 88% without sacrificing sensitivity, improving the area under the receiver operating characteristic curve (AUC) from 0.78 to 0.86 [11].

#### 3.3.2. Contrast-Enhanced Ultrasound (CEUS)

Contrast-enhanced ultrasound (CEUS) uses intravenous microbubble contrast agents to evaluate nodal perfusion patterns. In metastatic nodes, disruption of normal hilar vascular architecture leads to characteristic enhancement patterns, including heterogeneous enhancement, peripheral rim enhancement, and focal filling defects. A meta-analysis of 12 studies reported that CEUS had a pooled sensitivity of 89% and specificity of 84% for detecting nodal metastasis, outperforming conventional B-mode ultrasound [12]. The negative predictive value of CEUS was 92% in studies where only nodes with suspicious B-mode features underwent CEUS. More recently, CEUS has been explored for post-NAC assessment, with a study of 92 patients reporting a negative predictive value of 88% for predicting pathologic complete response [12].

#### 3.3.3. Shear-Wave Elastography (SWE)

Shear-wave elastography (SWE) quantifies tissue stiffness by measuring the propagation speed of shear waves induced by acoustic radiation force. Metastatic lymph nodes are typically stiffer than benign reactive nodes due to tumor infiltration and desmoplastic reaction. Using a cutoff of 28.5 kPa, SWE achieved a sensitivity of 83% and specificity of 85% for detecting nodal metastasis [13]. When combined with B-mode criteria, specificity increased to 92% while sensitivity remained above 80%. Notably, SWE measurements vary across equipment vendors, and standardization of measurement techniques remains an ongoing challenge.

### 3.4. Artificial Intelligence in Axillary Ultrasound

All AI models discussed in this section are currently classified as investigational; none have yet been incorporated into routine clinical guidelines. Recent developments in AI have brought axillary ultrasound to a new level of diagnostic sophistication. A deep learning model based on the DeiT-MLP-mixer architecture, trained on 8762 axillary ultrasound images, achieved an AUC of 0.89 for preoperative prediction of axillary lymph node metastasis [14]. The Noninvasive Lymph Node Staging (NILS) tool—an artificial neural network-based decision-support system—demonstrated an AUC of 0.87 for predicting axillary metastasis, with a negative predictive value of 93% for cN0 patients [15]. Use of NILS reduced the rate of unnecessary SLNB by 21% without increasing the false-negative rate. Complementary approaches using IoT-enabled deep transfer learning and hybrid machine learning have also demonstrated promise, though these have not been specifically validated for axillary assessment [16,17,18,19]. Notably, false-positive axillary findings on 18F-FDG PET-CT have been reported and should be interpreted with caution in the context of axillary staging [19].

Despite these promising results, several important limitations must be emphasized. Most AI studies in axillary ultrasound remain retrospective with inherent selection bias; external validation across different ultrasound machines, patient populations, and clinical settings remains limited. The black-box nature of deep learning models poses challenges for clinical interpretability. Prospective trials evaluating patient-centered outcomes are urgently needed [20]. Beyond the methodological limitations already discussed, several additional barriers hinder the routine clinical implementation of AI in axillary ultrasound. External validation across different ultrasound machines, patient populations, and clinical settings remains limited, and performance typically declines when models are applied to independent datasets. Dataset heterogeneity—including differences in image acquisition protocols, prevalence of node-positive disease, and annotation standards—further complicates model generalizability. Algorithm bias is a critical concern, as most AI models have been trained on datasets that may not adequately represent diverse patient demographics or disease presentations. Finally, regulatory approval pathways for AI-based medical devices remain complex and fragmented across jurisdictions; few AI tools have received clearance for routine clinical use in axillary staging. Addressing these challenges will require collaborative efforts in data sharing, prospective validation, and regulatory harmonization before AI can be reliably integrated into clinical workflows.

### 3.5. Practical Limitations and Quality Assurance in Axillary Ultrasound

Despite its widespread use, axillary ultrasound has important limitations affecting its reliability. Diagnostic performance is highly operator-dependent; interobserver agreement ranges from 0.60 to 0.80 for cortical thickness assessment. Equipment differences—including transducer frequency, beamforming algorithms, and preset optimization—can significantly affect image quality. Interpretation criteria (e.g., the 3 mm cortical thickness cutoff) have not been standardized across ultrasound systems. To mitigate these issues, we recommend structured training and credentialing for operators, standardized image-acquisition protocols, regular inter-observer audits, and incorporation of AI-based quality control tools.

## 4. Linking Ultrasound to Axillary De-Escalation: Why Imaging Matters

Before reviewing the surgical evidence, it is essential to understand the logical connection between ultrasound and axillary de-escalation. Without accurate preoperative nodal assessment, surgical de-escalation strategies cannot be safely implemented. Conversely, without the evidence from de-escalation trials, there would be little impetus to refine ultrasound techniques. These two domains are mutually reinforcing. The de-escalation trials discussed below—Z0011, AMAROS, OTOASOR, and SOUND—all relied on ultrasound as the gatekeeper for patient selection. Thus, ultrasound is not merely a diagnostic adjunct but the enabling technology that makes axillary de-escalation safe and feasible.

This section summarizes the evolution of axillary surgery from ALND to SLNB, contextualizing the role of ultrasound in enabling these changes.

## 5. Evolution of Axillary Management: From Radical Lymphadenectomy to Sentinel Lymph Node Biopsy

### 5.1. Historical Context: The Halsted Era and the Establishment of ALND

In the late 19th century, William Halsted introduced radical mastectomy with en bloc resection of the breast, pectoral muscles, and axillary lymph nodes, grounded in the theory that breast cancer spreads in a stepwise fashion—a model known as the Halstedian paradigm [2]. For nearly a century, ALND remained the standard of care. However, as understanding of breast cancer biology deepened, the Fisherian paradigm gradually gained acceptance, suggesting that breast cancer is a systemic disease from its outset and that extensive local surgery may not alter its natural history [21]. This conceptual shift laid the groundwork for a critical reassessment of the necessity of ALND.

ALND is associated with substantial postoperative morbidity. Long-term follow-up data indicate that the incidence of upper extremity lymphedema following ALND ranges from 20% to 30%, with additional complications including restricted shoulder mobility, nerve injury, pain, and seroma formation [22,23]. A large-scale, 23-year population-based study tracking the transition from ALND to SLNB demonstrated that while complication rates decreased significantly, long-term survival outcomes were not adversely affected [24]. Notably, advances in systemic adjuvant therapy have also profoundly influenced axillary surgical strategies, further undermining the rationale for routine ALND [21].

### 5.2. The Paradigm Shift: Development and Validation of Sentinel Lymph Node Biopsy

In the 1990s, Krag and colleagues and Giuliano and colleagues independently reported the application of SLNB in breast cancer [3]. ICG fluorescence-guided tracing has gained widespread clinical adoption due to its real-time visualization, absence of radiation exposure, and procedural simplicity [25]. Multiple validation trials have confirmed that in early-stage breast cancer patients with clinically negative axillae (cN0), the false-negative rate of SLNB can be maintained within 5–10% [26]. A comprehensive review confirmed that SLNB has now surpassed ALND as the predominant axillary staging modality [3], with the 23-year population study confirming stable breast cancer-specific mortality [24]. Furthermore, the 2026 updated guidelines from the AGO Breast Committee explicitly recommend SLNB as the standard axillary staging procedure for cN0 early breast cancer, with consideration of omitting SLNB in selected low-risk patients [26].

## 6. Evidence Base for Axillary De-Escalation Strategies

### 6.1. Landmark Clinical Trials: Z0011, Amaros, Otoasor, Sound, and Beyond

The driving force behind axillary surgical de-escalation has been a series of rigorously designed randomized controlled trials. The ACOSOG Z0011 trial enrolled patients with cT1–T2 breast cancer, 1–2 positive sentinel lymph nodes, and planned breast-conserving surgery with whole-breast irradiation, randomizing them to either ALND or SLNB alone. Long-term follow-up demonstrated no significant differences in overall survival or disease-free survival, while the ALND group experienced significantly higher rates of lymphedema and nerve injury [27,28]. It is important to emphasize that Z0011 enrolled only patients who underwent breast-conserving surgery with whole-breast irradiation; its applicability to mastectomy patients remains uncertain.

The AMAROS trial compared ALND with axillary radiotherapy (ART) in SLNB-positive patients. The 10-year results showed that axillary recurrence rates were exceptionally low in both arms (0.93% vs. 1.82%), meeting the non-inferiority criterion, with significantly lower lymphedema rates in the ART group [29]. A critical appraisal, however, highlights that ART includes comprehensive regional node irradiation, which carries its own morbidity profile. The OTOASOR trial similarly confirmed that regional nodal irradiation can safely replace ALND in carefully selected patients [4,5]. A multicenter Chinese cohort study reaffirmed the safety of omitting ALND in patients with 1–2 positive sentinel lymph nodes [30], and a propensity score-matched analysis further confirmed that omission of ALND with adjuvant regional nodal irradiation achieves comparable outcomes [31].

The SOUND trial enrolled patients with cN0 disease (confirmed by ultrasound) and small tumors (≤2 cm), comparing SLNB omission versus performance of SLNB. At a median follow-up of 5.2 years, the axillary recurrence rate was 0.5% in the SLNB omission group versus 0.4% in the SLNB group, meeting the prespecified non-inferiority criterion [32,33]. The INSEMA trial further demonstrated no significant difference in 5-year invasive disease-free survival between no SLNB, SLNB, and ALND arms in patients with T1–T2 tumors undergoing breast-conserving surgery [34]. Real-world validation studies have confirmed that omitting SLNB does not result in significant survival differences when SOUND/INSEMA criteria are applied [35,36,37].

The key evidence from these landmark trials is summarized in Table 2. Although these trials collectively support axillary de-escalation, their results must be interpreted strictly within their respective eligibility boundaries. The external validity to non-White or non-European populations remains under-explored. De-escalation strategies should be tailored to each patient’s individual risk profile, guideline recommendations, and available local expertise.

### 6.2. Omission of ALND in Mastectomy Patients with 1–2 Positive Sentinel Nodes

While the Z0011 trial established that completion ALND can be safely omitted in selected patients undergoing breast-conserving surgery with whole-breast irradiation, the applicability to mastectomy patients remains debated. A comprehensive review by Gentile and Tinterri systematically examined this question, concluding that emerging evidence supports the feasibility of omitting ALND in mastectomy patients with 1–2 positive sentinel nodes, though further randomized trials are needed [38]. Similarly, a study by Imoto et al. confirmed the feasibility of tailored axillary approaches in both cN0 and cN+ settings [39]. Clinicians should consider these data cautiously, pending further prospective validation and consensus guideline updates.

### 6.3. A Comparison of Traditional and US-Guided Axillary Pathways

Table 3 illustrates how ultrasound changes clinical practice by comparing the traditional versus US-guided pathways step by step.

This comparison highlights the central role of US in contemporary axillary management—from initial triage through post-treatment surveillance.

### 6.4. Lymph Node Management in the Neoadjuvant Chemotherapy Era

The increasing use of NAC in initially node-positive (cN+) patients presents both new challenges and opportunities. For patients who achieve axillary pathologic complete response (ypN0) after NAC, accurately assessing nodal status while minimizing surgical trauma has become a central clinical question [8]. International consensus guidelines recommend that for initially cN+ patients who convert to clinically node-negative after NAC, SLNB combined with TAD represents the key strategy for accurate axillary staging [8,9]. TAD involves marking the biopsy-proven metastatic node before NAC (via clip or carbon tattooing under US guidance), followed by combined SLNB and excisional biopsy of the marked node after NAC. A systematic review confirmed that TAD reduces the false-negative rate to below 5% [10].

Several technical considerations deserve emphasis. The false-negative rate of TAD is inversely related to the number of nodes retrieved; retrieval of three or more sentinel nodes plus the clipped node reduces the false-negative rate to <5% [8]. As demonstrated in the ACOSOG Z1071 trial, single-tracer SLNB after NAC yields a false-negative rate of approximately 10–15%, which decreases to <5% with dual tracers and retrieval of three or more sentinel nodes [42]. A nationwide Italian survey revealed that the transition from conventional ALND to TAD and SLNB in cN+ patients after NAC is accelerating [43]. A cross-sectional study confirmed that SLNB can safely replace ALND in patients who convert to cN0 after NAC [44].

With respect to systemic therapy, a pooled analysis of five randomized trials examined the interaction between LN surgery and CDK4/6 inhibitors, suggesting that novel systemic therapies may further reduce the intensity of axillary surgery required [45]. The AGO 2026 guidelines also recommend that for initially cN+ patients who achieve ypN0 after NAC, omission of ALND may be considered [26].

### 6.5. Unresolved Issues and Real-World Limitations

Management of residual nodal disease (ypN+) after NAC. The optimal strategy for patients with persistent axillary disease after NAC remains uncertain. Completion ALND is the traditional approach, but its benefit in patients receiving intensive adjuvant systemic therapy (e.g., T-DXd, CDK4/6 inhibitors) is increasingly debated. High-level evidence comparing ALND versus axillary radiotherapy in this subset is lacking. Ongoing trials such as the RNI Precision Trial are investigating optimized regional node irradiation strategies based on genomic risk assessment [46]. Outside of clinical trials, completion ALND (or axillary radiotherapy) remains the standard of care for ypN+ patients.

Applicability of SOUND/INSEMA findings to unselected populations. Both trials applied strict eligibility criteria (age >50 years, cT1, HR+/HER2−, grade 1–2, negative axillary ultrasound). The safety of SLNB omission in patients with borderline features (age <50, tumor size 2.1–2.5 cm, grade 3, or equivocal ultrasound findings) has not been validated. Extrapolation of trial results to such populations should be made with caution. Variations in international guidelines persist—AGO is more permissive of SLNB omission, whereas NCCN and ASCO remain more conservative [26,47]. Ultrasound limitations in obese patients and implementation challenges in low-resource settings also warrant attention.

## 7. Integrating Ultrasound with Molecular and Genomic Tools

### 7.1. Liquid Biopsy and ctDNA Monitoring

Liquid biopsy—particularly circulating tumor DNA (ctDNA) analysis—is attracting increasing attention for its potential role in axillary staging and treatment monitoring. ctDNA-guided axillary decision-making is currently exploratory and should be restricted to clinical research settings. Multiple studies have reported that ctDNA monitoring for predicting axillary pathologic complete response achieves sensitivity of 90–95% and specificity exceeding 95% in the post-NAC setting. As costs decline and standardization improves, ctDNA may become a more accessible tool for guiding axillary de-escalation. Integrating these molecular data with genomic assays such as the 21-gene recurrence score may further refine patient stratification for SLNB omission [48].

### 7.2. Multigene Profiling and Molecular Subtyping

The principles of precision medicine are fundamentally reshaping axillary lymph node management. For HR+/HER2− breast cancer, the necessity of routine intraoperative frozen section analysis of SLNB is being reassessed. Evidence suggests that in clinically node-negative HR+/HER2− patients, the clinical utility of intraoperative frozen section may be limited [49]. Genomic tools such as the 21-gene recurrence score are being explored for predicting the risk of axillary involvement, with Botty van den Bruele et al. demonstrating that nodal disease burden was not associated with elevated recurrence scores in post-menopausal women with clinically negative axillae [50].

The rapid evolution of targeted therapy is profoundly influencing axillary decision-making. The monarchE trial demonstrated that adjuvant abemaciclib significantly improved overall survival in high-risk HR+/HER2− breast cancer, with consistent benefit across patients with 1–3 nodes and those with four or more positive nodes, indicating that ALND is not required to determine CDK4/6 inhibitor candidacy [51]. In the HER2-low setting, T-DXd has demonstrated powerful systemic control over lymph node metastases. If a patient with 1–2 positive sentinel nodes and HER2-low status is planned for T-DXd therapy, the additional benefit of ALND is likely marginal.

It is important to note that while genomic assays are well-validated for systemic therapy decisions, their direct use for axillary surgical de-escalation remains off-label. Studies on SLN positivity rates after NAC using frozen section analysis provide additional stratification tools [52]. Traditional pathologic parameters—including tumor size, histologic grade, and Ki-67 index—remain important, with Nathanson et al. demonstrating low axillary metastasis rates in patients meeting specific pathologic criteria [40].

### 7.3. A Note on Guideline Comparisons

The role of ultrasound in axillary management varies across major guidelines. The NCCN (2025 v1) and ASCO guidelines both recommend SLNB for cN0 patients without explicit endorsement of SLNB omission [41,47]. The ESMO guidelines acknowledge the SOUND trial data, while the AGO 2026 guidelines go further, explicitly recommending that SLNB may be omitted in selected low-risk patients [26]. These differences reflect regional variations in the acceptance of US-based de-escalation strategies.

## 8. Toward a Multidisciplinary, Tiered Clinical Algorithm Integrating Ultrasound

Based on the evidence synthesized in this review, we propose a stepwise clinical algorithm structured into three tiers according to the strength of evidence. This algorithm represents the authors’ interpretation of the available evidence rather than a formal clinical practice guideline and should be applied in conjunction with local guidelines and individual patient preferences.

Tier 1–Current Standard of Care (Strong Evidence, Guideline-Endorsed)

- Preoperative axillary US with selective biopsy of suspicious nodes.
- US-guided clip placement for patients receiving NAC.
- SLNB for cN0 patients (or TAD for initially cN+ patients who convert to cN0 after NAC).
- Completion ALND or axillary radiotherapy according to residual nodal burden, with multidisciplinary discussion.

Tier 2–Guideline-Supported Options (Conditional, Based on Trial Eligibility)

- Omission of SLNB in patients meeting SOUND criteria (tumor ≤ 2 cm, negative axillary US, BCS + WBI).
- Axillary radiotherapy as an alternative to ALND in low-volume SLN-positive patients (AMAROS criteria).

Tier 3–Investigational Approaches (Not Yet Standard)

- ctDNA-guided post-NAC surgical decisions.
- Multigene assays to inform SLNB omission.
- Adjustment of axillary surgery based on planned adjuvant targeted therapies.

For all tiers, multidisciplinary discussion (surgery, radiology, pathology, medical oncology, radiation oncology) is strongly recommended at each decision point. The algorithm is a flexible guide, not a rigid prescription, and should be adapted to local resources, patient preferences, and individual clinical circumstances. A concise summary of these three tiers, categorizing each strategy by evidence level and clinical readiness, is provided in Table 4.

## 9. Future Directions

All the directions discussed below are at various stages of preclinical or early clinical development; they are presented as future research avenues rather than ready-for-practice tools.

### 9.1. Real-Time AI Interpretation

The development of real-time AI interpretation of axillary ultrasound has the potential to transform clinical practice. Current AI models operate in batch mode, requiring images to be uploaded and processed after acquisition. Real-time integration would provide immediate feedback to the operator, facilitating point-of-care decision-making [17]. Preliminary data from prototype systems suggest that real-time AI can achieve comparable diagnostic accuracy to offline models, with the additional advantage of reducing operator-dependent variability [17].

### 9.2. Imaging-Omics

Imaging-omics—the high-throughput extraction of quantitative features from medical images—has emerged as a promising approach for enhancing the predictive value of axillary ultrasound. By analyzing hundreds of texture, shape, and intensity features from US images, machine learning algorithms can identify subtle patterns that are not perceptible to the human eye. Preliminary studies have shown that radiomic features from axillary ultrasound can predict nodal metastasis with AUC values of 0.85–0.90 and may also predict molecular subtype and treatment response [18]. The integration of radiomic features with clinical and genomic data represents a frontier area of investigation.

### 9.3. Ultrasound-Guided Targeted Drug Delivery

Ultrasound-mediated microbubble destruction has been investigated as a strategy for targeted drug delivery to axillary lymph nodes. By loading therapeutic agents onto microbubbles and destroying them at the target site with focused ultrasound, it is theoretically possible to achieve high local drug concentrations with reduced systemic toxicity. Preclinical studies have demonstrated the feasibility of this approach in animal models of lymph node metastasis [19]. Clinical translation is still in early stages, but this approach may eventually offer a non-surgical therapeutic option for isolated nodal metastasis.

### 9.4. Integration into Clinical Practice

For these advances to translate into improved patient care, several challenges must be addressed. First, rigorous prospective validation of AI models and imaging-omics signatures is needed before they can be integrated into clinical workflows. Second, standardization of ultrasound acquisition protocols and image interpretation criteria is essential to ensure reproducibility across different equipment, operators, and patient populations. Third, the integration of advanced imaging tools into routine practice will require investment in training, infrastructure, and interdisciplinary collaboration. Fourth, ethical and regulatory frameworks must be developed to govern the use of AI in clinical decision-making, particularly with respect to accountability, transparency, and patient safety.

## 10. Conclusions

The management of axillary lymph nodes in breast cancer has undergone a remarkable transformation over the past three decades—from Halstedian radical clearance to precision-guided, multidisciplinary de-escalation. Ultrasound is now a key decision-support instrument within a multidisciplinary framework that integrates pathology, surgery, radiotherapy, and molecular oncology. Preoperative axillary ultrasound with selective biopsy is the standard of care for all early-stage breast cancer patients. US-guided clip placement enables TAD in patients receiving NAC, reducing post-NAC false-negative rates to <5%. Landmark de-escalation trials have progressively reduced axillary surgery, and ultrasound is essential for patient selection. Clinicians should actively adopt US-guided de-escalation strategies to minimize morbidity while ensuring oncological safety.

In summary, ultrasound-guided axillary de-escalation is currently supported by high-level evidence for the following patient populations: (1) patients with cT1-2 tumors and 1–2 positive sentinel nodes undergoing breast-conserving surgery with whole-breast irradiation (Z0011 criteria); (2) patients with cN0 disease and tumors ≤ 2 cm who meet SOUND criteria; and (3) initially node-positive patients who convert to cN0 after NAC and undergo TAD with dual-tracer localization. Conversely, the safety of de-escalation has not been established in patients undergoing mastectomy, those with higher nodal burden (≥3 positive nodes), patients with borderline or equivocal ultrasound findings, or those with residual nodal disease after NAC (ypN+). These populations require further prospective investigation, and management decisions should be guided by multidisciplinary discussion and individualized risk assessment.

## Figures and Tables

**Table 1:** Comparison of ultrasound techniques for axillary lymph node assessment.

Technique	Principle	Strengths	Limitations	Diagnostic Performance	Ideal Clinical Application
B-mode US	Gray-scale morphology (cortical thickness, hilum, shape)	Widely available, low cost, real-time, no radiation	Operator-dependent, moderate sensitivity (50–85%), interobserver variability	Pooled sensitivity 79%, specificity 78%	First-line screening and triage; biopsy guidance
CEUS	Microbubble contrast to assess perfusion patterns	Improved sensitivity (89%) and specificity (84%) over B-mode; helps distinguish reactive vs. metastatic	Requires contrast agent, longer exam, specialized expertise, lack of standardized criteria	Sensitivity 89%, specificity 84%; NPV 92% for problem-solving	Problem-solving tool for equivocal B-mode findings; post-NAC response assessment
SMI	Doppler-based slow-flow imaging of microvessels	High specificity (88%) without sacrificing sensitivity; visualizes peripheral vascularity	Equipment-dependent, limited multicenter validation	Sensitivity ~80%, specificity 88%	Reducing unnecessary biopsies in suspicious but equivocal nodes
SWE	Shear-wave speed to measure tissue stiffness	Objective quantitative metric; high NPV (89%) and improved specificity when combined with B-mode	Vendor-dependent cutoffs, transducer pressure and depth affect values; no universal threshold	Sensitivity 83%, specificity 85% (cutoff 28.5 kPa)	Adjunct to B-mode, especially in equivocal cases
3D-US	Volumetric acquisition with multiplanar reconstruction	Improves detection of focal cortical abnormalities; better spatial orientation	Longer acquisition and reconstruction time; limited clinical availability	Incremental sensitivity from 75% to 83%	Pre-surgical planning in selected cases (e.g., clip localization)
AI-assisted	Deep learning for automated detection and classification	High AUC (0.89); reduces interobserver variability; potential for non-invasive staging	Black-box nature, limited external validation, data-hungry, medicolegal concerns	AUC 0.89 (internal), 0.82 (external)	Research/adjunct; may eventually enable SLNB omission in low-risk patients

Abbreviations: US, ultrasound; CEUS, contrast-enhanced ultrasound; SMI, superb microvascular imaging; SWE, shear-wave elastography; AI, artificial intelligence; NPV, negative predictive value; AUC, area under the receiver operating characteristic curve.

**Table 2:** Summary of Key Evidence from Landmark Axillary De-escalation Trials.

Trial	Population	Intervention	Key Results	Key Limitations
Z0011	cT1-2, 1-2 SLN+, BCS + WBI	ALND vs. SLNB alone	No sig. diff. in OS/DFS; lower lymphedema in SLNB arm	BCS + WBI only; low proportion of high-risk patients; ultrasound protocol not standardized
AMAROS	cT1-2, SLN+, any surgery	ALND vs. ART (axillary RT)	10 yr axillary recurrence: 0.93% vs. 1.82%; less lymphedema with ART	ART toxicity not negligible; long-term cardiac/pulmonary effects under-reported
OTOASOR	cT1-2, SLN+ (low burden)	ALND vs. nodal RT	Regional RT safely replaces ALND; comparable survival	Smaller sample size; limited statistical power for rare events.
SOUND	cN0 (US-confirmed), small tumors (≤2 cm)	SLNB vs. no SLNB	SLNB omission feasible in stringently selected low-risk cN0 patients	Very strict inclusion criteria; short follow-up for late recurrences.
INSEMA	cN0, early BC	SLNB omission vs. SLNB	Ongoing; preliminary data support selective SLNB omission	Preliminary data; longer follow-up needed
Z1071	cN+, post-NAC	SLNB vs. ALND	FNR 10–15% (single tracer); <5% with dual tracer + ≥3 SLNs	Single-tracer FNR still >10%; dual tracer requires resource availability.

BCS = Breast-Conserving Surgery; WBI = Whole-Breast Irradiation; OS = Overall Survival; DFS = Disease-Free Survival; ART = Axillary Radiotherapy; cN0 = Clinically Node-Negative; FNR = False-Negative Rate.

**Table 3:** Stepwise Comparison of Traditional and US-Guided Axillary Management Pathways.

Step	Traditional Pathway	US-Guided (Contemporary) Pathway	Evidence/Key Trials
Initial assessment	Physical examination only	High-resolution axillary US	—
Node status determination	Based on clinical examination (palpation)	US morphology (cortical thickness >3 mm, loss of hilum)	[12,13,14,15]
Suspicious node management	SLNB or ALND directly	US-guided FNA/core biopsy + clip placement	[21,22,23]
Node-positive management	Upfront ALND	NAC with US-guided clip marking + post-NAC TAD	Z1071 [10,11,25]
Node-negative management	SLNB	SLNB or omission (SOUND criteria: tumor ≤2 cm, negative US, BCS + WBI)	SOUND [40,41]
Post-treatment surveillance	Clinical examination	Regular axillary US	—

**Table 4:** Categorization of axillary management strategies by level of evidence and clinical readiness.

Category	Definition	Key Strategies	Evidence Level	Clinical Recommendation
Established clinical practice	Widely adopted, guideline-endorsed, supported by high-level evidence	Preoperative axillary US with selective biopsy; US-guided clip placement before NAC; SLNB for cN0 patients; TAD for cN+ patients who convert to cN0 after NAC	High (multiple RCTs, meta-analyses, international guidelines)	Recommended as standard of care
Guideline-supported approaches	Supported by trial data, endorsed by some guidelines, but conditional on patient selection	Omission of SLNB in patients meeting SOUND criteria (age > 50, cT1, HR+/HER2−, grade 1–2, negative US, BCS + WBI); Axillary RT as alternative to ALND in low-volume SLN-positive patients (AMAROS criteria)	Moderate (landmark RCTs with strict eligibility criteria)	Recommended in carefully selected patients; shared decision-making required
Investigational technologies	Promising but lacking prospective validation; not yet guideline-endorsed	ctDNA-guided surgical decisions; AI-assisted axillary US; multigene assay-guided SLNB omission; ultrasound-guided targeted drug delivery	Low (retrospective, single-center, or preclinical studies)	Should be restricted to clinical trials or research settings

US, ultrasound; NAC, neoadjuvant chemotherapy; SLNB, sentinel lymph node biopsy; cN0, clinically node-negative; cN+, clinically node-positive; TAD, targeted axillary dissection; BCS, breast-conserving surgery; WBI, whole-breast irradiation; RT, radiotherapy; ALND, axillary lymph node dissection; ctDNA, circulating tumor DNA; AI, artificial intelligence; RCT, randomized controlled trial.

## Data Availability

No new data were created or analyzed in this study. Data sharing is not applicable to this article.

## Supplementary Materials

The following supporting information can be downloaded at: https://www.mdpi.com/article/10.3390/cancers18152405/s1, Figure S1. PRISMA 2020 flow diagram illustrating the literature search and study selection process for this narrative review. The diagram summarizes the identification, screening, and inclusion process following the Preferred Reporting Items for Systematic Reviews and Meta-Analyses (PRISMA) 2020 guidelines. A total of 2847 records were identified from three electronic databases (PubMed, Web of Science, and Scopus) using predefined search terms. After removal of duplicate records, 2224 records were screened by title and abstract, of which 1986 were excluded. The remaining 238 full-text reports were sought for retrieval; 31 could not be accessed. A total of 207 full-text reports were assessed for eligibility, and 154 were excluded based on predefined criteria. Ultimately, 53 studies met the inclusion criteria and were included in this narrative review.
