# Supplementary material for: Ultrasound-Guided Axillary Management in Early-Stage Breast Cancer: From Diagnosis to De-Escalation
Source: Cancers (Basel). 2026 Jul 26;18(15):2405. doi: 10.3390/cancers18152405 (PMC13465595; doi:10.3390/cancers18152405)
Supplement: Supplementary file 1 [file cancers-18-02405-s001.zip › cancers-4430844-supplementary.pdf]

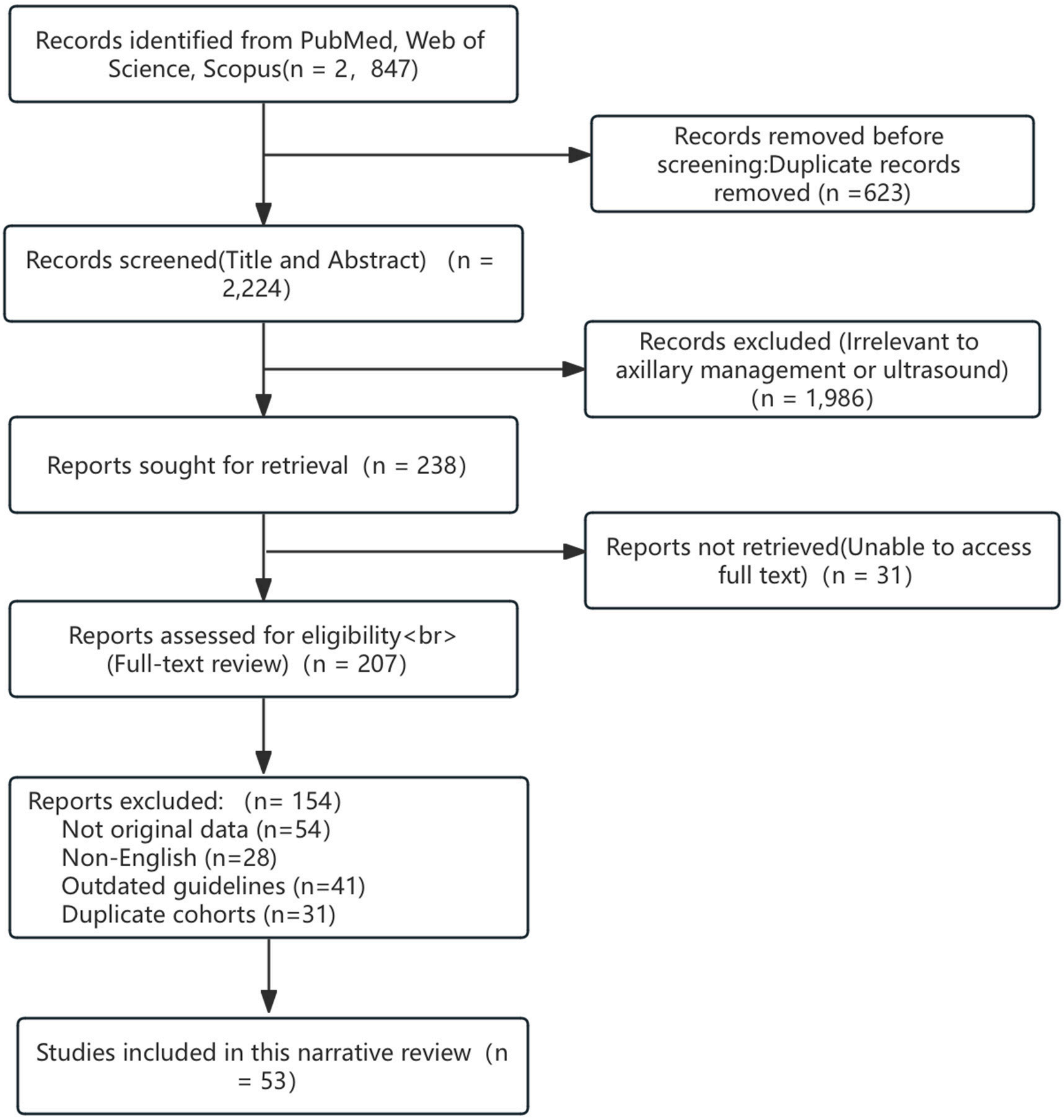

Supplementary Figure S1. PRISMA 2020 flow diagram illustrating the literature search and study selection process for this narrative review. The diagram summarizes the identification, screening, and inclusion process following the Preferred Reporting Items for Systematic Reviews and Meta-Analyses (PRISMA) 2020 guidelines. A total of 2847 records were identified from three electronic databases (PubMed, Web of Science, and Scopus) using predefined search terms. After removal of duplicate records, 2224 records were screened by title and abstract, of which 1986 were excluded. The remaining 238 full-text reports were sought for retrieval; 31 could not be accessed. A total of 207 full-text reports were assessed for eligibility, and 154 were excluded based on predefined criteria. Ultimately, 53 studies met the inclusion criteria and were included in this narrative review.
